# Cyclosporine A in the treatment of dry eye syndrome in patients with Sjögren’s disease—a systematic review

**DOI:** 10.1016/j.ero.2025.11.006

**Published:** 2025-12-10

**Authors:** Jarryl H.J. Tsai, Jonathan T.W. Au Eong, Kah-Guan Au Eong

**Affiliations:** 1 Lee Kong Chian School of Medicine, Nanyang Technological University, Singapore, Singapore; 2 Singapore General Hospital, Department of General Surgery, Singapore, Singapore; 3 International Eye Cataract Retina Centre, Mount Elizabeth Medical Centre and Farrer Park Medical Centre, Singapore, Singapore; 4 Department of Ophthalmology and Visual Sciences, Khoo Teck Puat Hospital, Singapore, Singapore

## Abstract

**Objectives:**

The objective of this study is to systematically evaluate the efficacy and safety of cyclosporine A (CsA) in treating dry eye syndrome (DES) among patients with Sjögren’s disease (SjD).

**Methods:**

A systematic search was conducted in PubMed, Cochrane, Embase, CINAHL, and Web of Science on January 30, 2025, for randomised controlled trials (RCTs) assessing CsA for SjD-related DES. Studies were included if they evaluated CsA in any concentration, formulation, or mode against placebo or vehicle controls and reported at least 1 of the following: Schirmer test, tear breakup time (TBUT), Rose Bengal staining score, Ocular Surface Disease Index (OSDI), or adverse events. Risk of bias was assessed using the Cochrane Risk of Bias 2 tool.

**Results:**

Four RCTs met inclusion criteria. Three of them investigated topical formulations (0.05%-2.0%), while 1 studied oral CsA. Topical CsA was associated with significant improvements in TBUT, Rose Bengal staining, and OSDI scores compared with controls. Schirmer test results were mixed, with 2 of 3 topical CsA studies showing significant improvement. Oral CsA showed no significant benefit in Schirmer test. Adverse effects were mild and consistent with known profiles, with systemic side effects occurring only in the oral CsA study. Two studies had high risk of bias, and 2 had some concerns.

**Conclusions:**

Topical CsA appears effective and well-tolerated for treating DES in SjD, particularly in improving tear film stability and ocular surface integrity. Oral CsA remains of uncertain benefit. Further large-scale, high-quality RCTs are warranted.

**WHAT IS ALREADY KNOWN ON THIS TOPIC**

• While topical cyclosporine (CsA) is widely used for dry eye syndrome (DES), there has been no systematic review specifically assessing the efficacy of CsA in patients with DES associated with Sjögren disease (SjD).

**WHAT THIS STUDY ADDS**

• This study suggests that topical CsA improves tear breakup time, Rose Bengal staining scores, and Ocular Surface Disease Index in patients with DES associated with SjD. Oral CsA shows no benefit and has more systemic adverse effects.

**HOW THIS STUDY MIGHT AFFECT RESEARCH, PRACTICE OR POLICY**

• This study supports current European League Against Rheumatism guidelines on the use of topical CsA for SjD-associated DES and highlights the limited role of oral CsA. Clinicians can more confidently use topical CsA in this group of patients.

Alt-text: Unlabelled box

## INTRODUCTION

Dry eye syndrome (DES) is a prevalent condition that significantly impairs quality of life, with symptoms ranging from ocular discomfort to visual disturbances [1]. Among the various aetiologies of DES, Sjögren’s disease (SjD) stands out as a systemic autoimmune disorder characterised by focal lymphocytic sialadenitis [2], leading to severe aqueous tear deficiency and pronounced DES.

While the efficacy of cyclosporine A (CsA) in treating DES in the general population is well-known [[3], [4], [5]], there is a notable lack of focused analyses on its effectiveness specifically within the patient population with SjD. Given the distinct pathophysiology of SjD-related dry eye, it is imperative to assess whether CsA’s immunomodulatory properties offer comparable benefits in this subgroup. This study aimed to systematically evaluate the efficacy and safety of CsA in managing DES among patients with SjD, thereby providing targeted insights for clinicians treating this condition.

## METHODS

### Search strategy

A systematic search was done across PubMed, Cochrane, Embase, CINAHL, and Web of Science databases on January 30, 2025. There was no language or publication date restrictions. The primary search strategy was built in PubMed using a combination of Medical Subject Headings and natural language related to ‘cyclosporine’, ‘dry eye syndrome’, and ‘Sjögren’s syndrome’. Boolean operators were used to combine concepts, and filters were applied to include only randomised controlled trials (RCTs). The search strategy was then adapted to the other databases.

### Eligibility criteria

The eligibility criteria for this systematic review were defined to ensure a focused and clinically relevant assessment of CsA for DES associated with SjD. Only RCTs were included in this study. The population criteria included patients with a confirmed diagnosis of SjD-related DES, while studies of patients with other ocular surface diseases were excluded. The intervention of interest was CsA in any concentration, formulation, or route of administration, whereas combination therapies involving CsA were excluded. Comparators included placebo and empty vehicles. Eligible studies reported at least 1 of the following outcomes: Schirmer test score, tear breakup time (TBUT), Rose Bengal staining score, Ocular Surface Disease Index (OSDI), or adverse events.

### Study selection

The study selection process followed the Preferred Reporting Items for Systematic reviews and Meta-Analyses (PRISMA) guidelines [6] and was conducted in accordance with the predefined eligibility criteria. Two independent investigators (JHJT and JTWAE) screened all retrieved records in a 2-stage process: title/abstract screening, followed by full-text review. Any discrepancies were resolved through discussion.

### Data extraction

The data extraction process was conducted systematically to ensure consistency and accuracy. Data were extracted using a predefined data extraction form. Key information included study characteristics, participant details, intervention/comparator information, and reported outcomes. Extracted data were then organised into standardised tables for synthesis and analysis, ensuring a structured approach to evaluation.

### Risk of bias

The risk of bias (RoB) for included RCTs was assessed using the Cochrane Risk of Bias 2 (RoB 2) tool. This tool evaluates the following 5 domains: (1) bias arising from the randomisation process, (2) bias due to deviations from intended interventions, (3) bias due to missing outcome data, (4) bias in measurement of the outcome, and (5) bias in selection of the reported result. The overall RoB judgement was determined according to the criteria outlined in the RoB 2 guidelines.

## RESULTS

### Screening process

**Figure 1:**
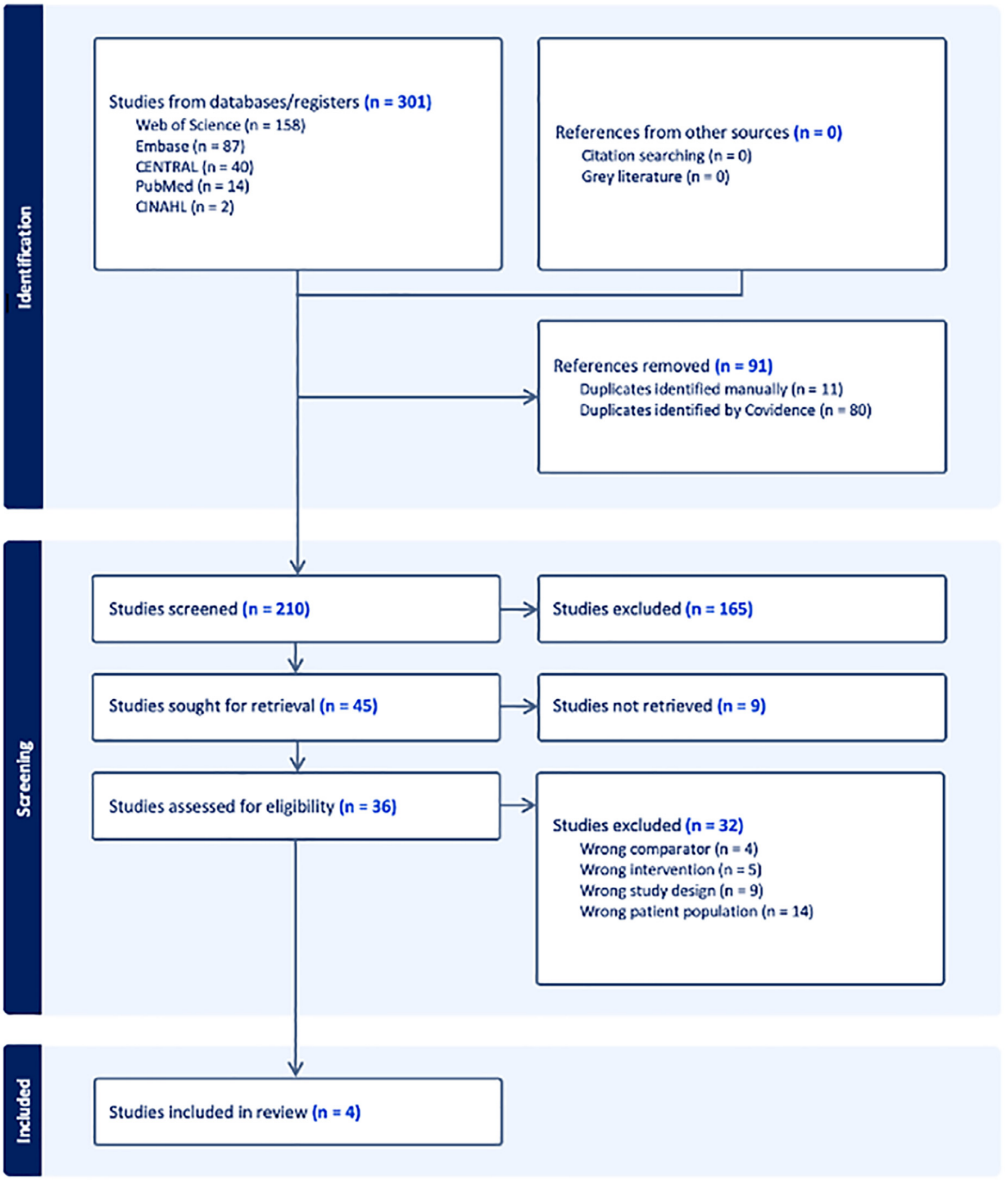
Preferred Reporting Items for Systematic reviews and Meta-Analyses (PRISMA) diagram.

The search flow is illustrated in a PRISMA diagram (Fig 1). The initial search yielded 301 studies, of which 91 were duplicates. The remaining 210 studies were screened according to the eligibility criteria, of which 165 studies were removed due to irrelevance. The remaining 45 studies were to be retrieved for full-text review, but 9 studies were unable to be retrieved. The remaining 36 studies underwent a full-text review, and finally, 4 studies were included in the final study.

### Study characteristics

The characteristics of the 4 studies are shown in Table 1 [[7], [8], [9], [10]]. A total of 130 patients were included over a treatment duration between 2 and 6 months. The baseline Schirmer scores ranged from 3.50 to 7.40 mm. All studies explicitly stated that participants were not receiving concurrent systemic immunosuppressive therapy, except for that by Fan et al [9], in which this detail was not specified. Three of these studies used topical CsA ranging from 0.05% to 2%, while one study used oral CsA. The results from the last follow-up of the studies, which ranged from 2 to 6 months, were analysed. For all studies, there was no significant difference reported between the intervention and control populations, including parameters such as age, sex/gender (as reported in respective articles), as well as baseline TBUT, Rose Bengal staining score, Schirmer test, and OSDI results.

**Table 1:** Characteristics of studies

Reference, year	Country	Sample size		Age (y), mean ± SD		Sex/gender (male:female)		Type of intervention		Type of control		Treatment duration (mo)
		Intervention	Control	Intervention	Control	Intervention	Control	Intervention name	Dosage, daily	Control name	Dosage, daily	
Drosos et al [7], 1986	Greece	10	10	47.3 ± 8.4	54.0 ± 11.2	1:9	0:10	Oral CsA	5 mg/kg body weight	Placebo	—	6
Gündüz and Ozdemir [8], 1994	Turkey	15	15	—	—	—	—	Topical CsA (2.0%)	4 times	Olive oil vehicle	4 times	2
Fan et al [9], 2003	Taiwan	24	16	50.8 ± 10.7	53.5 ± 11.5	2:22	1:15	Topical CsA (0.1%)	Twice	Castor oil vehicle	Twice	3
Gao et al [10], 2023	China	20	20	51.4 ± 12.4	58.4 ± 9.55	2:18	1:19	Topical CsA (0.05%)	Twice	Hyaluronic acid sodium eye drops	4 times	3

CsA, cyclosporine A.

### RoB assessment

**Figure 2:**
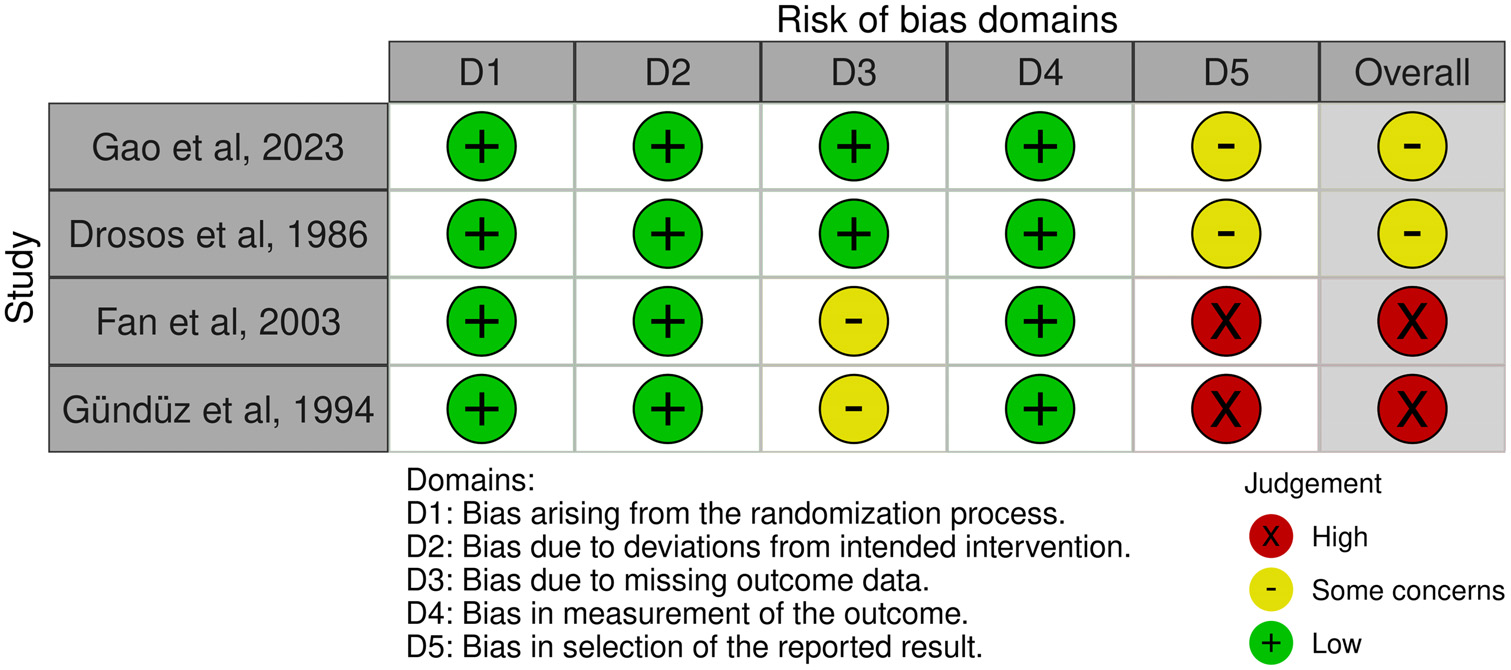
Risk of bias assessment of the included studies.

The RoB 2 assessment for the included RCTs is summarised in Figure 2 [[7], [8], [9], [10]]. Two studies [7,10] were judged to have an overall RoB categorised as ‘some concerns’, primarily due to potential bias in the selection of the reported results (D5). Both studies demonstrated a low RoB across other domains. In contrast, the studies by Fan et al [9] and Gündüz and Ozdemir [8] were rated as having a high overall RoB. These studies showed serious concerns in missing outcome data (D3) and selection of reported results (D5), potentially affecting the reliability of their findings. Given these assessments, the evidence from the included studies should be interpreted with caution, particularly when considering studies with a high RoB.

### Outcome measures

#### Schirmer test

All 4 studies used Schirmer test as a measure of effectiveness of CsA (Table 2) [[7], [8], [9], [10]]. Two studies reported that topical CsA performed significantly better than the control [9,10], while 1 did not [8]. There was no significant difference between oral CsA and placebo in improving Schirmer test results (*P* values not reported).

**Table 2:** Data on Schirmer test

Reference, year	Schirmer test (mm), mean ± SD					
	Baseline			Final		
	Control	Intervention	*P*	Control	Intervention	*P*
Drosos et al [7], 1986	7.40 ± 5.30	4.80 ± 6.20	—	5.00 ± 5.1	5.20 ± 10.60	Not significant
Gündüz and Ozdemir [8], 1994	5.42 ± 0.62	5.63 ± 0.82	>.05	5.50 ± 0.72	5.85 ± 0.92	>.05
Fan et al [9], 2003	3.50 ± 0.97	3.38 ± 0.09	—	—	—	.007
Gao et al [10], 2023	4.35 ± 2.54	5.55 ± 3.63	.133	4.40 ± 3.28	8.15 ± 4.34	<.001

#### Tear breakup time

Two studies that investigated TBUT found a significant increase in TBUT in patients who used topical CsA compared with that in those who were given placebo [8,10].

#### Rose Bengal staining score

Two studies that measured Rose Bengal staining scores reported a significant decrease in Rose Bengal staining score in patients using topical CsA compared with the score in those using placebo (*P* = .007; *P* < .01) [8,9].

#### Ocular Surface Disease Index

The sole study that investigated OSDI reported a significant decrease in OSDI (lower score indicates less severe dry eye symptoms and a lesser impact on quality of life) in patients using topical CsA compared with that in the control group (*P* < .001) [10].

#### Adverse effects

Adverse effects reported differed significantly between studies using topical and those using oral CsA. Patients using topical CsA reported mild ocular discomfort, severe stinging, running nose, tearing, diffuse punctate lesions, and local allergic reaction [[8], [9], [10]]. For oral CsA, the effects included hirsutism, infections, hypertension, gingival hypertrophy, nausea, and vomiting [7].

## DISCUSSION

### Summary of findings

This systematic review of 4 RCTs included 3 investigating topical CsA (0.05%-2.0%) and 1 evaluating oral CsA. Topical treatment was generally associated with improvements in TBUT, Rose Bengal staining scores, and OSDI compared with placebo or vehicle controls. Schirmer test results for topical CsA were inconsistent, with 2 of 3 studies reporting significant improvement. Oral CsA showed no significant benefit in Schirmer test. Adverse events with topical therapy were mostly mild and ocular in nature, whereas oral therapy was associated with systemic side effects.

### Methods of evaluation

The studies used 4 main tests to evaluate the potential benefits of CsA for DES in SjD. These tests assess key aspects of DES, providing both objective and subjective measures of disease severity and treatment efficacy. Schirmer test measures tear production by assessing the amount of aqueous tears secreted over a set time, which is particularly relevant in SjD, where autoimmune-mediated lacrimal gland dysfunction is the cause of dry eye. TBUT is a tool widely used by many clinicians, including researchers in the Sjogren’s International Collaborative Clinical Alliance, to measure tear film stability [11] Rose Bengal staining score assesses ocular surface damage and recovery in DES [12]. OSDI measures patient-reported symptoms, acting as a subjective measure of the severity of DES [13].

### Topical CsA

Three RCTs found topical CsA of varying concentrations to be effective across multiple outcome measures [[8], [9], [10]]. While Schirmer test results were mixed, with 1 study reporting no significant difference [8] and others showing improvement [9,10], other indicators of dry eye severity demonstrated more robust positive effects. Both studies assessing TBUT found a significant increase in tear film stability with topical CsA (*P* < .001; *P* < .01) [8,10], indicating improved ocular surface hydration. Similarly, the reduction in Rose Bengal staining scores reported by Fan et al [9] (*P* = .007) and Gündüz and Ozdemir [8] (*P* < .01), suggests a protective effect on the ocular surface, further supporting the efficacy of CsA in treating DES associated with SjD. Additionally, the sole study measuring OSDI showed a significant reduction in symptoms (*P* < .001) [10], reinforcing the potential symptomatic relief provided by topical CsA. Together, these findings demonstrate the benefits of topical CsA in treating DES associated with SjD, aligning with existing research that supports its efficacy across all forms of DES [[3], [4], [5]].

### Oral CsA

The only RCT using oral CsA showed no significant improvement in Schirmer test and did not assess other key parameters such as TBUT, Rose Bengal staining score, or OSDI [7]. Oral CsA, while having a broader immunosuppressive effect, lacks consistent evidence for direct ocular benefit. It is therefore unsurprising that the European League Against Rheumatism recommendations for managing SjD emphasise the use of topical CsA for DES treatment, without endorsing systemic (oral) CsA for this indication [14].

### Adverse effects of CsA

The adverse effects of topical CsA in patients with SjD were similar to those observed in individuals using topical CsA for DES in the general population [15]. Common side effects included ocular burning, stinging, redness, and blurred vision, which are well-documented with CsA eye drops. These symptoms are typically mild and transient.

Oral CsA was associated with typical side effects well-documented in systemic CsA therapy, including hirsutism, increased risk of infections, hypertension, gingival hypertrophy, nausea, and vomiting [16,17]. This similarity suggests that the tolerability profile of both topical and oral CsA in patients with SjD aligns with its broader use in managing DES.

### Limitations

Several limitations of this systematic review should be acknowledged. First, the number of included studies was small, and variations in CsA formulation, concentration, and treatment duration may have contributed to the heterogeneity in results. Second, not all studies reported on all key outcome measures, particularly patient-reported symptoms (eg, OSDI), limiting comprehensive conclusions on the symptomatic benefits of CsA. Third, information on concurrent systemic immunosuppressive therapy was available for only 3 studies, all of which confirmed no such use [7,9,10]. This information was not reported by Fan et al [9], which limits comparability across trials. Fourth, the diagnostic and classification criteria for SjD have evolved over time, introducing potential bias in patient selection across studies. In addition, Gündüz and Ozdemir [8] included only patients with secondary SjD, further contributing to heterogeneity among the small study cohorts. Further large-scale, high-quality RCTs in this area are therefore warranted.

## Conclusion

In conclusion, topical, but not oral CsA, shows potential benefits in improving tear film stability, ocular surface integrity, and OSDI in patients with SjD and DES. Larger, rigorously designed RCTs with standardised diagnostic criteria, consistent follow-up durations, and transparent reporting of both positive and negative outcomes are needed to better establish the long-term efficacy and safety of CsA in treating DES in patient with SjD.

## CRediT authorship contribution statement

**Jarryl H.J. Tsai:** Writing – review & editing, Writing – original draft, Data curation, Conceptualization. **Jonathan T.W. Au Eong:** Writing – review & editing, Conceptualization. **Kah-Guan Au Eong:** Writing – review & editing, Conceptualization.

## Appendix Supplementary materials

Image, application 1
